# Regional differences in the effects of healthy aging on depressive symptoms: a Korean longitudinal study of aging (2006–2020)

**DOI:** 10.3389/fpubh.2024.1256368

**Published:** 2024-01-16

**Authors:** Soo Jin Kang, Jinseub Hwang, Dohyang Kim, Bongjeong Kim

**Affiliations:** ^1^Department of Nursing, Daegu University, Daegu, Republic of Korea; ^2^Department of Statistics, Daegu University, Gyeongsan, Cheongju-si, Republic of Korea; ^3^Department of Nursing, Cheongju University, Cheongju-si, Republic of Korea

**Keywords:** healthy aging, middle-aged adults, urban population, depressive symptoms, rural population

## Abstract

**Background:**

Depression is a widely prevalent, often recurrent condition. To analyze the regional differences in depressive symptoms over time, we investigated urban–rural differences in change in depression over time in South Korea and the association between healthy aging and depressive symptoms among middle-aged and older adults.

**Methods:**

Data collected in the Korean Longitudinal Study of Aging, from 2006 to 2020, of adult participants aged ≥45 years without depressive symptoms were analyzed. Healthy aging was defined under five principal components: absence of chronic disease, good physical function, normal cognitive function, active social engagement, and good psychological adaptation. Depressive symptoms were measured using the short version of the Center for Epidemiologic Studies Depression Scale. Using the Andersen-Gill model for recurrent time-to-event, we examined the effect of healthy aging on depressive symptoms, with a subgroup analysis based on the residential area.

**Results:**

Of the 7,708 participants, 78.2% lived in urban areas and 39.4% achieved healthy aging. In 2008, rural residents had a higher incidence of depressive symptoms (rural 11.8%; urban 8.9%); however, after 2016, the depressive symptoms of urban residents gradually increased (rural 6.4%; urban 12.1%). Unhealthy aging (adjusted hazard ratio = 3.04, 95% confidence interval: 2.72–3.39) and urban residence (adjusted hazard ratio = 1.15, 95% confidence interval: 1.06–1.24) were risk factors for depressive symptoms. The subgroup analysis revealed that individuals who did not achieve healthy aging had an increased risk of depressive symptoms, regardless of their residential area (hazard ratio [95% confidence interval]: urban, 3.13 [2.75–3.55]; rural 2.59 [2.05–3.28]).

**Conclusion:**

As urbanization accelerates, urban residents have a higher risk of depressive symptoms than rural residents. Healthy aging is an essential factor in reducing depressive symptoms. To achieve healthy aging, appropriate interventions and policies that target the middle-aged adults and gradually extend to older adults are needed, considering individual and regional factors.

## 1 Introduction

With the advancement in medical technology and the consequent extension of life expectancy, aging has become one of the major public health challenges worldwide (1, 2). South Korea has the fastest-aging population globally (3). In 2017, more than 14% of the Korean population was in the ≥65 year-age group, and this proportion increased to 16.6% in 2022 (4). Depression among older adults is an important risk factor for suicide in many countries (5) and diminishes the quality of life (6). The suicide rate in Korea has remained the highest among the Organization for Economic Co-operation and Development (OECD) countries since 2013 (7). Notably, the suicide rate of the older adult population is approximately 2.26 times that of Korea’s other age groups. The middle-aged group (40–64 years) has the second highest suicide rate (8), underscoring the mental health needs of both the middle-aged and older adults.

Approximately 280 million people worldwide have depression (9), and 20–35% of older adults in Korea have experienced depression (10), with 27.8% of the total population with depression in Korea being in their 40s or 50s (11). Depression is a highly recurrent health problem, with 60% of those who recover from the first episode being at risk of additional episodes and approximately 70% of those with a history of two episodes having another recurrence (12). However, most previous studies investigating the prevalence of depressive symptoms and associated factors have used cross-sectional designs (13–15). Consequently, the studies are inherently limited to establish a causal relationship or demonstrate consistent associations with depressive symptoms.

The World Health Organization (WHO) posited the concept of healthy aging, defined as “the process of developing and maintaining the functional ability that enables wellbeing in older age.” Health aging is not a new concept, but rather, an inclusive concept related to successful, active, and optimized aging (16). Healthy aging decreases the risks of requiring long-term care (17) and all-cause mortality (18). Due to the large proportion of older adults in Korea, healthy aging has increasingly garnered focus, and previous reports on the relationship between healthy aging and depression indicate that unhealthy aging is a predictor of a higher risk of depression (19, 20). In particular, most studies using the Korean Longitudinal Study on Aging (KLoSA) – a nationally representative sample of Koreans older adults – explored the factors that influence depression (21, 22). Individual factors, including women, having a lower income, living alone, consuming alcohol, and being visually impaired, are known risk factors for increased depressive symptoms (10, 13, 14, 23). Recent studies have reported differences in the neighborhood effect on depression (24, 25). These neighborhood-level factors include infrastructural, interactive social, and demographic characteristics of residents, as well as political and environmental characteristics (26). Residential areas (urban/rural) affect inhabitants in various contexts, especially older adults versus young adults (27). Hwang and Kim (24) reported that urban residents have lower depressive symptoms in Korea in association with spatial distribution patterns, as urban areas have various facilities, such as healthcare services, employment opportunities, transportation, and social capital. However, the opposite results have been reported previously (28). Studies and resources on the relationship between depressive symptoms and neighborhood-level variables in Korea are scarce, and the existing studies are small-sized convenience samples (14) or cross-sectional studies (29, 30) that do not reflect changes in the differences over time. To generate robust results by recognizing that depressive symptoms do not end as a single event and recur subsequently, we applied a new statistical model – the Anderson–Gill model (31) – which is effective wherein the event of interest occurs more than once in a participant, for example, the incidence of depression after injury or readmission outcome analysis (32, 33). In contrast to previous studies that focused on the prevalence of depressive symptoms and the influencing factors, this study is meaningful as it expanded the focus on the incidence of recurrent depressive symptoms using longitudinal data.

**Figure 1:**
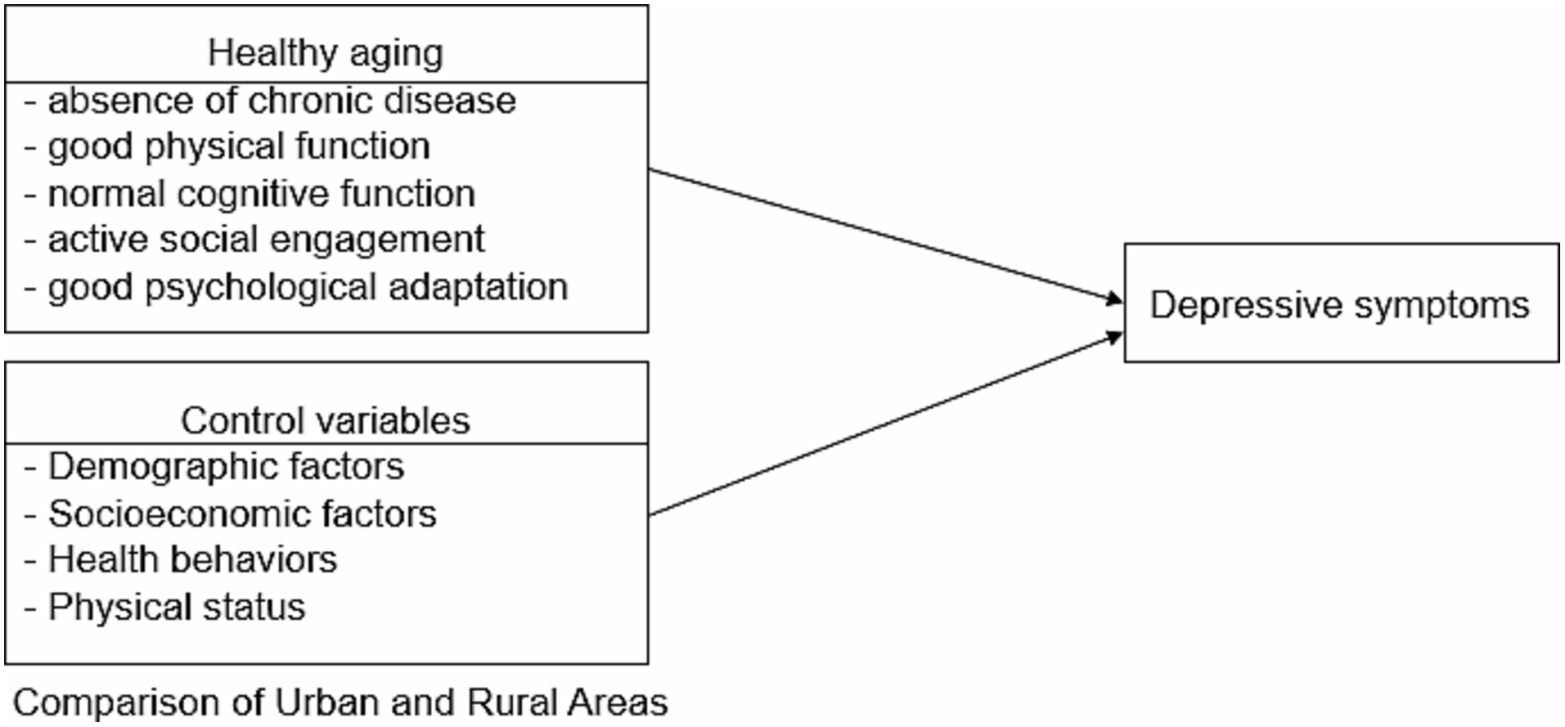
Conceptual framework.

This study aimed to (1) compare how depressive symptoms change over time in urban and rural areas in Korea; (2) assess whether regional differences in the effects of healthy aging on depressive symptoms in Korean adults vary over time; and (3) assess whether risk factors for depressive symptoms differ between urban and rural areas over time (Figure 1).

*Hypotheses 1:* The incidence of depressive symptoms significantly differs between urban and rural areas.

*Hypotheses 2:* Health aging and factors affecting the incidence of depression differ between urban and rural areas from 2006 to 2020 in Korea.

## 2 Methods

### 2.1 Study design and population

**Figure 2:**
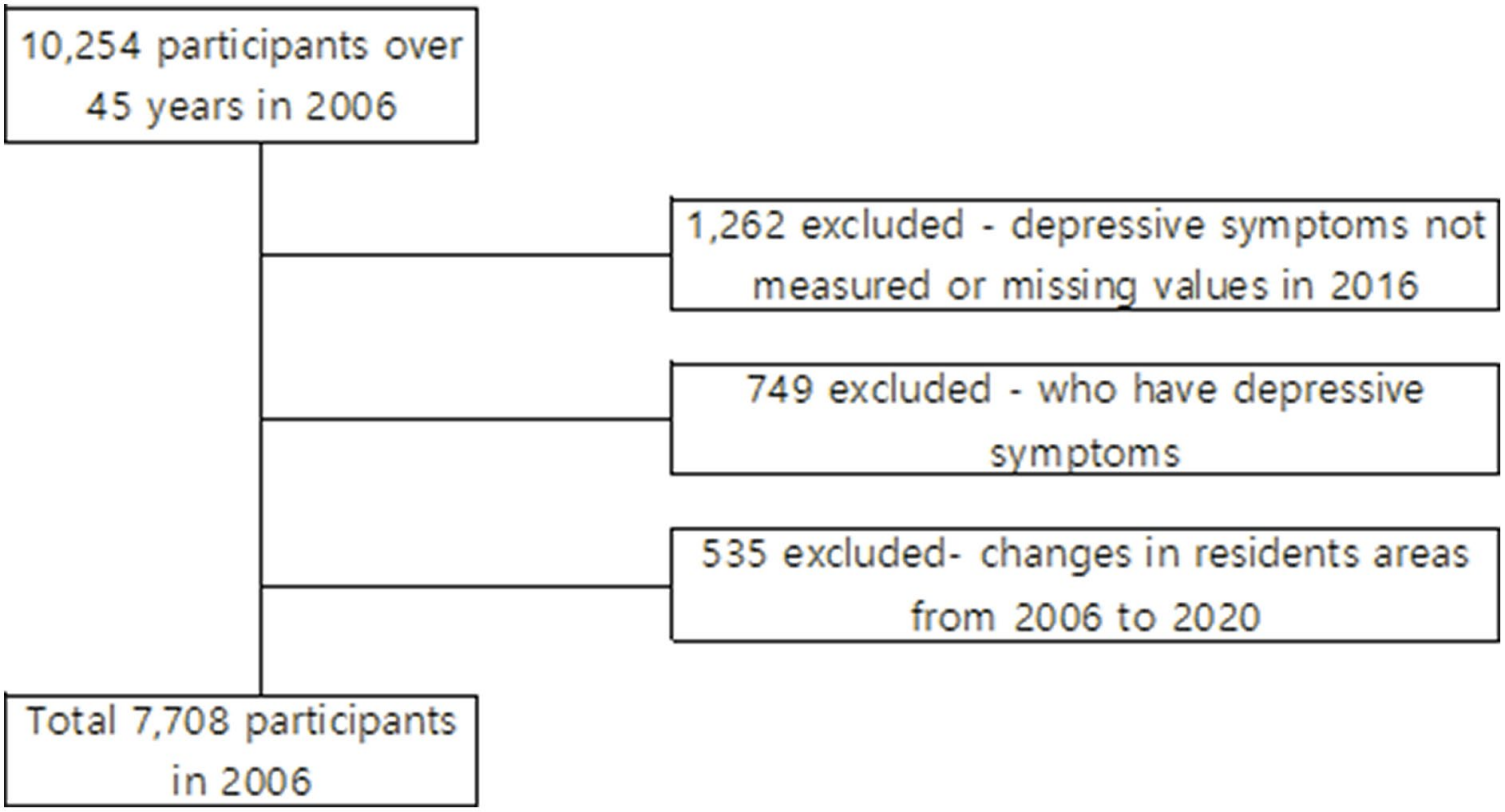
Participants selection process. Flowchart of the study participants from 2006 to 2020.

This is a secondary data analysis study using longitudinal data. Data for this study were derived from the 1st to the 8th waves of the KLoSA, from 2006 to 2020. KLoSA is an ongoing, longitudinal, nationwide study conducted by the Labor Institute of the Korean Ministry of Labor once every 2 years. It is a panel survey of community-dwelling people aged ≥45 years at the time of the baseline interview in 2006. The participants were randomly recruited using multilevel stratified sampling based on geographical areas and housing types representing the entire population of South Korea. In the 2006 baseline survey, the original panel interviewed 10,245 participants. All participants took part in a computer-assisted personal interview. Detailed information on the KLoSA survey and design can be found on its website.^1^ The target population of the present study were individuals aged ≥45 years. We excluded individuals who had incomplete data or missing values (*n* = 1,262), those who had depressive symptoms (Center for Epidemiologic Studies Depression Scale [CES-D-10] ≥4) in wave 1 (*n* = 749), and those who had changes in their residence between urban and rural areas (*n* = 535). Finally, 7,708 participants were included in the baseline evaluation, as shown in the flowchart of participants and selection process (Figure 2).

### 2.2 Variables and measurements

#### 2.2.1 Outcome variables

The main outcome of this study was the incidence of depressive symptoms, which were measured using the CES-D-10. The CES-D-10 comprises 10 items listed in the original 20-item version and has been validated (34). The Korean version of the CES-D-10 was validated by Shin (35) and showed good internal consistency (Cronbach’s alpha = 0.79). For this study, Cronbach’s alpha was 0.83. The total score is 10 points; higher scores indicate more severe depressive symptoms. We used a cutoff score of 4 points based on previous studies (36). If the CES-D-10 score was ≥4, participants were considered to have depressive symptoms, which constitute an event (depressive symptoms).

#### 2.2.2 Independent variables

##### 2.2.2.1 Healthy aging

As healthy aging is a multidimensional concept, we used the operational definition from literature reviews (37–39). Healthy aging is a binary variable equal to 1 if the following five dimensions are considered: (1) absence of chronic disease, (2) good physical function, (3) normal cognitive function, (4) active social engagement, and (5) good psychological adaptation. Respondents who satisfied all the five domains were classified as a healthy aging group, whereas the remaining respondents were classified as an unhealthy aging group. The five domains are defined below:

1. Absence of chronic disease: Healthy aging refers to the absence of the following five diseases, which are the major causes of death in Korea: cancer, heart disease, chronic lung disease, diabetes mellitus, and cerebrovascular disease (number of diseases = 0) (38).
2. Good physical function: To determine good physical function, activities of daily living (ADLs) and instrumental activities of daily living (IADLs) were used. ADLs include dressing, continence, indoor transfer, eating, toilet use, and showering, whereas IADLs include grooming, ability to use the telephone, shopping, food preparation, housekeeping, driving or using public transportation, medication use, handling finances, doing laundry, and managing medications. If participants had no disability in ADLs (ADL = 0) and not more than one disability in IADL (IADL = 0 or 1), they were considered to have healthy aging (38).
3. Normal cognitive function: Cognitive function was measured using the Korean Mini-Mental Status Examination (MMSE-K), and total scores ranged from 0 to 30, with higher scores indicating better cognitive function (40). We classified respondents with a score ≥ 24 as having a normal cognitive function (38). Cronbach’s alpha for MMSE-K at the time of development was 0.84 (41) and 0.76 in this study.
4. Active social engagement: Healthy aging involves participation in social activities, including religious activities, leisure activities/sports, alumni events, volunteering, political events, and nongovernmental organization events. Active social engagement was defined as engaging in one or more of the abovementioned activities (social activities >1) (39).
5. Good psychological adaptation: This was measured as the mean satisfaction with health, economic status, marital status, relationship with children, and general life on a scale of 0 to 100. Respondents with a mean score ≥ 60 were classified as having good psychological adaptation (39).

##### 2.2.2.2 Control variables

Participants’ demographics, socioeconomic status, health behaviors, and physical status were considered independent variables. Demographic variables included sex and age (45–54, 55–64, >65 years). Socioeconomic factors included marital status (married/ unmarried/divorced/widowed), education level (elementary or lower school, middle school, high school, and college or higher), household income level (low/middle-low/middle-high/high), insurance type (national health/medical benefit), contact with children (frequently engaged with at least one child/frequently engages with all children/ occasionally engages with all children/ rarely engages with all children), contact with neighbors (less than once a week/more than once a week), and labor force participation (active/inactive). Health behavioral factors included smoking (current/past/never), alcohol consumption (yes/no), regular physical activity (yes/no), and regular diet (yes/no). Physical status factors included body mass index (BMI; underweight/normal/overweight), vision (good/poor), and hearing (good/poor). BMI was classified as overweight (BMI ≥25 kg/m^2^), normal (18.5 ≤ BMI <25 kg/m^2^), or underweight (BMI <18.5 kg/m^2^) according to the WHO parameters for Asian adults (42).

##### 2.2.2.3 Urban and rural areas

The urban and rural areas are divided according to the administrative districts in Korea. The “dong” is a unit district of large cities or provinces. “Metropolitan cities,” “provinces,” “cities,” and “dongs” are defined as urban areas, whereas “eups” and “myeons” are defined as rural areas in this country. The KLoSA provided the codebook with the classified residential areas.

### 2.3 Statistical analysis

First, the general characteristics of the study population, healthy aging, depression, and control variables are presented using descriptive statistics, that is, frequencies and percentages for categorical variables. The chi-square test was used to compare the general characteristics, control variables, and healthy aging components between the urban and rural areas at baseline. Second, we compared the incidence rates of depressive symptoms from 2006 to 2020 between the urban and rural areas. Third, the Andersen–Gill model was applied to investigate the effect of healthy aging on the incidence of depressive symptoms over time. Participants in the KloSA were assessed for depressive symptoms and not for the diagnosis of depressive disorder because it was assumed that present depressive symptoms would not persist until the following assessment 2 years later. The Andersen–Gill model is the most frequently applied model for recurrent time-to-event data and is a simple extension of the Cox proportional hazards regression model (30, 31). It is based on the assumption that the instantaneous risk of experiencing an event at a time since study induction remains the same, regardless of whether previous events have occurred (43, 44). In this study, the dependent variable was depression status (1 = depressive symptom, CES-D-10 ≥ 4). Independent variables were healthy aging, regional areas, and control variables. Depressive symptoms could be repeated several times during the eight follow-up assessments conducted during the study period. In Model 1 (unadjusted), the incidence of depressive symptoms was determined according to independent variables and healthy aging components from 2008 to 2020. In Model 2, the adjusted effect was identified by adding healthy aging and control variables in Model 1. In Model 3, subgroup analysis was performed to identify regional differences in factors related to depressive symptoms. We calculated crude and adjusted hazard ratios (HR), 95% confidence interval (CI), and *p*-values. All statistical analyses were performed using SAS version 9.4 (SAS Institute Inc., Cary, NC, United States), and *p*-values <0.05 were considered to indicate statistical significance.

### 2.4 Ethical statement

The original data are publicly available free of charge from the KLoSA website, published by the Korea Employment Information Service.^2^ This study used a deidentified secondary dataset. Therefore, it was exempted from review by our Institutional Review Board (1040621-202202-HR-E001).

## 3 Results

### 3.1 General characteristics and healthy aging between urban and rural areas

Table 1 shows the baseline characteristics of the participants. In total, 7,708 participants were included (3,520 males and 4,188 females). Among them, 78.2% (6,028) lived in urban areas. With regard to individual and social factors, 54.3% of the participants were female. Approximately 43.1% of all participants had an education level below elementary school, 39.1% assessed their household income as above middle-high, 57.4% had an inactive labor force status, and 41.3% responded that they rarely had contact with their children. Regarding health behaviors, approximately 60–70% of participants were nonsmokers or nondrinkers; however, 59.7% did not exercise regularly. Regarding health status, most (96.6%) participants had normal BMI. Between participants from urban and rural areas, there were significant differences in age, education level, household income level, insurance, frequency of contact with neighbors or children, participation in the labor force, regular exercise or diet, BMI, vision, and hearing.

**Table 1:** Baseline characteristics of the participants (*N* = 7,778).

Variables	Category	Total	Urban	Rural	*p*-value
Number of participants		7708 (100.0)	6028 (100.0)	1680 (100.0)	–
Sex	Men	3520 (45.7)	2752 (45.7)	768 (45.7)	0.965
	Women	4188 (54.3)	3276 (54.4)	912 (54.3)	
Age (years)	45–54	2703 (35.1)	2335 (38.8)	368 (21.9)	<0.0001
	55–64	2214 (28.7)	1732 (28.7)	482 (28.7)	
	>65	2791 (36.2)	1961 (32.5)	830 (49.4)	
Education	Elementary	3319 (43.1)	2225 (36.9)	1094 (65.1)	<0.0001
	Middle school	1333 (17.3)	1074 (17.8)	259 (15.4)	
	High school	2208 (28.7)	1951 (32.4)	257 (15.3)	
	College	848 (11.0)	778 (12.9)	70 (4.2)	
Marital status	Married	6345 (82.3)	4976 (82.6)	1369 (81.5)	0.314
	Unmarried/divorced/widowed	1363 (17.7)	1052 (17.5)	311 (18.5)	
Household income	High	2134 (27.7)	1629 (27.0)	505 (30.1)	<0.0001
	Middle-high	880 (11.4)	613 (10.2)	267 (15.9)	
	Middle-low	1994 (25.9)	1461 (24.2)	533 (31.7)	
	Low	2700 (35.0)	2325 (38.6)	375 (22.3)	
Insurance type	National	7552 (98.0)	5916 (98.1)	1636 (97.4)	0.050
	Medical benefit	156 (2.0)	112 (1.9)	44 (2.6)	
Contact with children	Frequently engaged with at least one child	2348 (30.5)	1781 (29.6)	567 (33.8)	<0.0001
	Frequently engages with all children	401 (5.2)	325 (5.4)	76 (4.5)	
	Occasionally engages with all children	1776 (23.0)	1232 (20.4)	544 (32.4)	
	Rarely engages with all children	3,183 (41.3)	2,690 (44.6)	493 (29.4)	
Contact with neighborhood (number of times)	<1/week	2633 (34.2)	2225 (36.9)	408 (24.3)	<0.0001
	≥1/week	5075 (65.8)	3803 (63.1)	1272 (75.7)	
Labor force participation	Active	3283 (42.6)	2491 (41.3)	792 (47.1)	<0.0001
	Inactive	4425 (57.4)	3537 (58.7)	888 (52.9)	
Smoking	Never	5445 (70.6)	4277 (71.0)	1168 (69.5)	0.502
	Past	759 (9.9)	590 (9.8)	169 (10.1)	
	Current	1504 (19.5)	1161 (19.3)	343 (20.4)	
Regular physical activity	Yes	3110 (40.4)	2743 (45.5)	367 (21.9)	<0.0001
	No	4598 (59.7)	3285 (54.5)	1313 (78.2)	
Alcohol consumption	Yes	3060 (39.7)	2405 (39.9)	655 (39.0)	0.501
	No	4648 (60.3)	3623 (60.1)	1025 (61.0)	
Regular diet	Yes	7098 (92.1)	5505 (91.3)	1593 (94.8)	<.0001
	No	610 (7.9)	523 (8.7)	87 (5.2)	
BMI (kg/m^2^)	Underweight: <18.5	265 (3.4)	185 (3.1)	80 (4.8)	0.001
	Normal: 18.5–24.9	7443 (96.6)	5843 (96.9)	1600 (95.2)	
	Overweight: ≥25.0	0 (0.0)	0 (0.0)	0 (0.0)	
Hearing	Poor	2106 (27.3)	1568 (26.0)	538 (32.0)	<0.0001
	Good	5602 (72.7)	4460 (74.0)	1142 (68.0)	
Vision	Poor	4989 (64.7)	3813 (63.3)	1176 (70.0)	<0.0001
	Good	2719 (35.3)	2215 (36.8)	504 (30.0)	

BMI, body mass index.

Table 2 presents the distribution of healthy aging and its components according to the urban and rural areas in the baseline population. In general, 39.4% of the participants were considered healthy aging, including 41.0% of those in urban areas and 33.8% of those in rural areas, with a significant difference between living areas at baseline (*p* < 0.0001). Regarding the five domains of health aging, good psychological adaptation was only achieved in 64.6% (lowest) of the population, whereas 97.2% (highest) had good physical function. There were significantly higher levels of physical function, cognitive function, and active social participation in urban than in rural areas.

**Table 2:** Description of the components of healthy aging at baseline (*N*=7,778).

Variables			Total	Urban	Rural	*p*-value
HA			3039 (39.4)	2471 (41.0)	568 (33.8)	<0.0001
Unhealthy aging			4669 (60.6)	3557 (59.0)	1112 (66.2)	
Components	Definition	Category				
Absence of chronic disease	Number of chronic					
	0	Yes	6523 (84.6)	5112 (84.8)	1411 (84.0)	0.412
	≥ 1	No	1185 (15.4)	916 (15.2)	269 (16.0)	
Good physical function	ADL					
	0	Yes	7492 (97.2)	5870 (97.4)	1622 (96.6)	0.068
	≥1	No	216 (2.8)	158 (2.6)	58 (3.5)	
	IADL					
	0–1	Yes	7094 (92.0)	5576 (92.5)	1518 (90.4)	0.004
	≥2	No	614 (8.0)	452 (7.5)	162 (9.6)	
	Sum of ADL and IADL					
	0–1	Yes	7065 (91.7)	5553 (92.1)	1512 (90.0)	0.006
	≥2	No	643 (8.3)	475 (7.9)	168 (10.0)	
Normal cognitive function	MMSE-K					
	≥24	Yes	6090 (79.0)	4931 (81.8)	1159 (69.0)	<0.0001
	<24	No	1618 (21.0)	1097 (18.2)	521 (31.0)	
Active social engagement	Participation in one or more activities					
	≥1	Yes	5712 (74.1)	4557 (75.6)	1155 (68.8)	<0.0001
	0	No	1996 (25.9)	1471 (24.4)	525 (31.3)	
Good psychological adaptation	Satisfaction with life					
	≥60	Yes	4979 (64.6)	3894 (64.6)	1085 (64.6)	0.991
	<60	No	2729 (35.4)	2134 (35.4)	595 (35.4)	

ADL, activities of daily living; IADL, instrumental activities of daily living; MMSE-K, Mini-Mental State Examination; HA, healthy aging.

### 3.2 Incidence of depressive symptoms from 2006 to 2020

**Figure 3:**
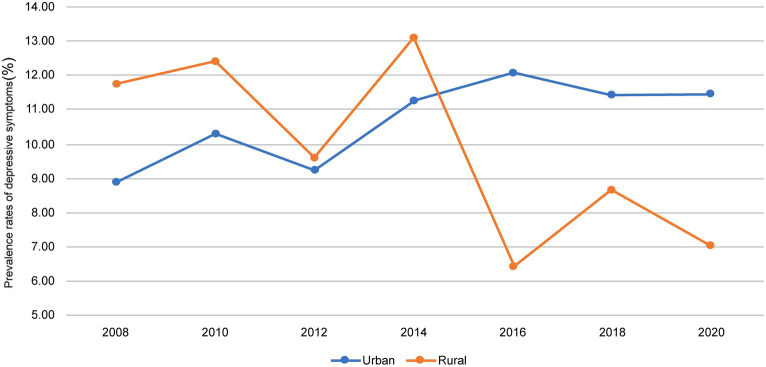
Changes in the incidence of depressive symptoms from 2008 to 2020.

Figure 3 shows the incidence of depressive symptoms between urban and rural areas from 2008 (2nd wave) to 2020 (8th wave). At the beginning of 2008, the incidence of depressive symptoms was higher in rural (11.8%) than in urban areas (8.9%). In rural areas, the incidence of depressive symptoms showed a strong downward trend in 2020 compared to that in 2008, despite fluctuations. After the 6th wave, the incidence of depressive symptoms was significantly higher in urban than in rural areas (6th: 12.1% vs. 6.4%, *p* < 0.001; 7th: 11.4% vs. 8.6%, *p* = 0.009; 8th: 11.5% vs. 7.0%, *p* < 0.001; Supplementary Table S1).

### 3.3 Effects of healthy aging on depressive symptoms from 2006 to 2020

Table 3 shows the factors related to depressive symptoms and their HRs among all participants (from 2008 to 2020) using time-dependent Cox regression in the crude and adjusted models. In the crude model, the region was not a significant factor for depressive symptoms (HR = 1.03, 95% CI: 0.95–1.10); however, in the adjusted model, the effect of the region (urban) on depressive symptoms was significant (HR = 1.15, 95% CI: 1.06–1.24). Furthermore, unhealthy aging had a significant effect on depressive symptoms (adjusted HR = 3.04). All five components of health aging (number of chronic diseases, ADLs, IADLs, MMSE-K, and participation in one or more activities) were significantly associated with depressive symptoms (Supplementary Table S2). Additionally, lower educational levels (elementary and middle school), higher household income level, medical benefits, poor contact with neighbors, inactive labor force status, no alcohol consumption, lower weight, poor vision, and poor hearing were significant factors for depressive symptoms.

**Table 3:** Factors related to depressive symptoms.

	Variables	Crude		*p*-value	Adjusted		*p*-value
		HR	95% CI		HR	95% CI	
Region	Urban	1.03	(0.95–1.10)	0.505	1.15	(1.06–1.24)	0.001
	Rural	1.00	[ref]		1.00	[ref]	
Sex	Men	1.00	[ref]		1.00	[ref]	
	Women	1.29	(1.21–1.38)	<0.0001	1.00	(0.91–1.11)	0.950
Age (years)	45–54	1.00	[ref]		1.00	[ref]	
	55–64	0.99	(0.46–0.52)	<0.0001	0.90	(0.83–0.98)	0.010
	> 65	1.71	(1.31–2.22)	<0.0001	0.88	(0.67–1.15)	0.334
Education	Elementary	2.77	(2.41–3.19)	<0.0001	1.31	(1.12–1.52)	0.001
	Middle school	1.67	(1.43–1.96)	<0.0001	1.23	(1.05–1.44)	0.012
	High school	1.29	(1.11–1.50)	0.001	1.14	(0.98–1.33)	0.098
	College	1.00	[ref]		1.00	[ref]	
Marital status	Married	1.00	[ref]		1.00	[ref]	
	Unmarried/divorced/widowed	1.77	(1.65–1.91)	<0.0001	1.07	(0.98–1.16)	0.131
Household income	High	1.97	(1.81–2.14)	<0.0001	1.22	(1.12–1.33)	<0.0001
	Middle-high	1.89	(1.71–2.10)	<0.0001	1.24	(1.12–1.39)	<0.0001
	Middle-low	1.50	(1.37–1.63)	<0.0001	1.14	(1.05–1.25)	0.003
	Low	1.00	[ref]		1.00	[ref]	
Insurance type	National	1.00	[ref]		1.00	[ref]	
	Medical benefit	1.67	(1.41–1.98)	<0.0001	1.19	(1.00–1.41)	0.045
Contact with children	Frequently engaged with at least one child	1.16	(1.07–1.26)	0.000	1.01	(0.93–1.09)	0.902
	Frequently engages with all children	0.85	(0.72–1.00)	0.053	0.97	(0.82–1.15)	0. 699
	Occasionally engages with all children	1.19	(1.10–1.28)	<0.0001	1.06	(0.98–1.14)	0.150
	Rarely engages with all children	1.00	[ref]		1.00	[ref]	
Contact with neighborhood (number of times)	<1/week	2.01	(1.89–2.14)	<0.0001	1.80	(1.68–1.92)	<0.0001
	≥1/week	1.00	[ref]		1.00	[ref]	
Labor force participation	Active	1.00	[ref]		1.00	[ref]	
	Inactive	2.70	(2.50–2.92)	<0.0001	1.67	(1.53–1.83)	<0.0001
Smoking	Never	1.36	(1.22–1.50)	<0.0001	1.02	(0.90–1.15)	0.788
	Past	1.31	(1.16–1.48)	<0.0001	1.10	(0.97–1.24)	0.150
	Current	1.00	[ref]		1.00	[ref]	
Regular physical activity	Yes	1.00	[ref]		1.00	[ref]	
	No	1.73	(1.61–1.86)	<0.0001	1.33	(1.23–1.43)	<0.0001
Alcohol consumption	Yes	1.00	[ref]		1.00	[ref]	
	No	1.92	(1.78–2.08)	<0.0001	1.24	(1.14–1.35)	<0.0001
Regular diet	Yes	1.00	[ref]		1.00	[ref]	
	No	1.93	(1.75–2.13)	<0.0001	1.67	(1.51–1.85)	<.0001
BMI (kg/m^2^)	Underweight: <18.5	2.22	(1.97–2.50)	<0.0001	1.31	(1.16–1.49)	<0.0001
	Normal: 18.5–24.9	1.00	[ref]		1.00	[ref]	
Hearing	Poor	2.59	(2.42–2.76)	<0.0001	1.49	(1.39–1.61)	<0.0001
	Good	1.00	[ref]		1.00	[ref]	
Vision	Poor	2.86	(2.59–3.16)	<0.0001	1.50	(1.34–1.66)	<0.0001
	Good	1.00	[ref]		1.00	[ref]	
HA	All HA	1.00	[ref]		1.00	[ref]	
	Unhealthy aging	5.18	(4.66–5.75)	<0.0001	3.04	(2.72–3.39)	<0.0001

HR, hazard ratio; CI, confidence interval; BMI, body mass index; HA, healthy aging.

### 3.4 Effects of health aging on depressive symptoms according to the residential areas

Table 4 shows the results of the subgroup analysis. Throughout the study period (2006–2020), a lower educational level (elementary school) was associated with depressive symptoms in rural areas. Household income level significantly affected depressive symptoms in urban areas, while in rural areas, its effects were mixed. In urban areas, the groups above the mid-low household income level were more likely to report increased depressive symptoms than the low-income group. However, in rural areas, depressive symptoms were not significant in the middle-high household income group. Depressive symptoms showed a higher incidence in those having less frequent contact with their neighbors than in those with frequent contact in both regions (urban HR = 1.87, 95% CI: 1.74–2.02; rural HR = 1.49, 95% CI: 1.30–1.71). Participation in the labor force decreased the risk of depressive symptoms, whereas there was a higher risk for depressive symptoms in economically inactive (urban HR = 1.67, 95% CI: 1.50–1.85; rural HR = 1.67, 95% CI: 1.42–1.97) than in economically active participants.

**Table 4:** Regional comparison of the time-dependent Cox recurrent models between depressive symptoms and related factors.

Variables	Categories	Urban				Rural	
		HR	95% CI	*p*-value	HR	95% CI	*p*-value
Sex	Men	1.00	[ref]		1.00	[ref]	
	Women	1.04	(0.93–1.16)	0.532	0.92	(0.76–1.13)	0.428
Age (years)	45–64	1.00	[ref]		1.00	[ref]	
	65–74	0.88	(0.80–0.96)	0.005	0.95	(0.80–1.12)	0.522
	75–84	1.07	(0.95–1.20)	0.267	1.04	(0.86–1.26)	0.666
	>85	0.80	(0.57–1.11)	0.185	1.09	(0.69–1.73)	0.717
Education	Elementary	1.33	(1.13–1.56)	0.001	1.06	(0.68–1.64)	0.801
	Middle school	1.18	(0.99–1.40)	0.064	1.25	(0.79–1.96)	0.343
	High school	1.16	(0.98–1.36)	0.078	0.87	(0.54–1.39)	0.548
	College	1.00	[ref]		1.00	[ref]	
Marital status	Married	1.00	[ref]		1.00	[ref]	
	Unmarried/divorced/widowed	1.04	(0.95–1.15)	0.390	1.09	(0.91–1.30)	0.356
Household income	High	1.16	(1.05–1.27)	0.004	1.37	(1.12–1.67)	0.002
	Middle-high	1.32	(1.17–1.49)	<0.0001	1.06	(0.83–1.34)	0.661
	Middle-low	1.28	(1.16–1.42)	<0.0001	0.73	(0.59–0.91)	0.004
	Low	1.00	[ref]		1.00	[ref]	
Insurance type	National	1.00	[ref]		1.00	[ref]	
	Medical benefit	1.12	(0.91–1.38)	0.281	1.32	(0.96–1.81)	0.083
Contact with children	Frequently engaged with at least one child	0.99	(0.90–1.09)	0.805	1.09	(0.93–1.29)	0.298
	Frequently engages with all children	0.94	(0.78–1.13)	0.507	1.18	(0.80–1.73)	0.403
	Occasionally engages with all children	1.09	(1.00–1.20)	0.046	0.93	(0.79–1.09)	0.347
	Rarely engages with all children	1.00	[ref]		1.00	[ref]	
Contact with neighborhood (number of times)	<1/week	1.87	(1.74–2.02)	<0.0001	1.49	(1.30–1.71)	<0.0001
	≥1/week	1.00	[ref]		1.00	[ref]	
Labor force participation	Active	1.00	[ref]		1.00	[ref]	
	Inactive	1.67	(1.50–1.85)	<0.0001	1.67	(1.42–1.97)	<0.0001
Smoking	Never	0.99	(0.86–1.13)	0.840	1.14	(0.88–1.49)	0.327
	Past	1.08	(0.94–1.24)	0.294	1.15	(0.88–1.51)	0.294
	Current	1.00	[ref]		1.00	[ref]	
Regular physical activity	Yes	1.00	[ref]		1.00	[ref]	
	No	1.39	(1.28–1.51)	<0.0001	0.98	(0.81–1.17)	0.794
Alcohol consumption	Yes	1.00	[ref]		1.00	[ref]	
	No	1.21	(1.09–1.33)	<0.0001	1.34	(1.13–1.60)	0.001
Regular diet	Yes	1.00	[ref]		1.00	[ref]	
	No	1.72	(1.54–1.92)	<0.0001	1.56	(1.22–2.00)	0.001
BMI (kg/m^2^)	Underweight: <18.5	1.41	(1.22–1.63)	<0.0001	1.16	(0.93–1.46)	0.198
	Normal: 18.5–24.9	1.00	[ref]		1.00	[ref]	
Hearing	Poor	1.58	(1.46–1.72)	<0.0001	1.23	(1.05–1.42)	0.008
	Good	1.00	[ref]		1.00	[ref]	
Vision	Poor	1.44	(1.28–1.62)	<0.0001	1.64	(1.29–2.08)	<0.0001
	Good	1.00	[ref]		1.00	[ref]	
HA	All HA	1.00	[ref]		1.00	[ref]	
	Unhealthy aging	3.13	(2.75–3.55)	<0.0001	2.59	(2.05–3.28)	<0.0001

HR, hazard ratio; CI, confidence interval; BMI, body mass index; HA, healthy aging.

Regional comparison of depressive symptoms and related factors between urban and rural areas revealed that health aging lowered the risk of depressive symptoms. In both residence areas, participants with unhealthy aging had a higher risk of depressive symptoms (urban HR = 3.13, 95% CI: 2.75–3.55; rural HR = 2.59, 95% CI: 2.05–3.28) than those with healthy aging. Regarding health behaviors, regular diet decreased the risk of depressive symptoms in both areas, and participants who did not perform regular physical activities showed a significantly increased risk of depressive symptoms in urban areas alone (HR = 1.39, 95% CI: 1.28–1.51). Poor vision and hearing increased the risk of depressive symptoms, regardless of the residence area.

## 4 Discussion

This study conducted an analysis of longitudinal data from 2006 to 2020 to further understand the relationship between health aging and depressive symptoms, focusing specifically on regional differences in Korea. The adjusted analysis showed that the incidence of depressive symptoms was significantly higher in urban areas than in rural areas, and the risk of depressive symptoms was 3.04 times higher in unhealthy adults than in those with healthy aging.

Differences in depressive symptoms among individuals from urban and rural areas have been an interesting research topic. Previous studies investigating the prevalence of depressive symptoms in urban and rural areas have reported inconsistent results (15, 28, 45). China Family Panel Studies examined 8,025, 7,808, and 4,887 older adults aged 60 years and above in 2016,2018, and 2020 and found that rural residents had a higher prevalence of depressive symptoms than urban residents (46). In contrast, a cross-sectional study of a cohort from the Canadian Longitudinal Study on Aging, which analyzed 21,241 adults aged 45–85 years from 2010 to 2014, showed that rural and peri-urban residents had lower scores of depressive symptoms than urban residents (47). Similarly, in a systematic review and meta-analysis that compared residential areas (48), urban residents had a significantly higher prevalence of depressive symptoms than rural residents in developed countries, although this trend was not observed in developing countries. Purtle et al. (48) attributed the regional differences in depressive symptoms to urbanization, wherein urbanization increased the risk of mental health problems. Several studies have reported that urbanization is associated with the prevalence of depressive symptoms (45, 49). Urban residents are more likely to have lower social support (49), and be exposed to stressors related to urban environments, including artificial light at night, neighborhood crime, traffic congestion, and noise (48). In addition to environmental pollution, rapid urbanization has increased other public health issues, such as chronic and mental health diseases and changes in people’s living and working habits, all of which have resulted in adverse health effects (50).

Globally, with increasing urbanization, more than 55% of the world’s population lived in urban areas in 2018, and by 2050, the urbanized population is expected to rise to two-thirds of the overall population (51). Korea is one of the few countries that has rapidly progressed from a low-income to a high-income country and has thereby experienced rapid economic development and urbanization. At the baseline of this study, 78.2% of the cohort lived in urban areas. In 1970, the urbanization rate in Korea was 50.2% (52), rising to 90.7% in 2008 and 91.3% in 2021 (53). Therefore, the sample of this study, KLoSA, is reasonable and not biased. In Korea, many previous studies have compared levels of depressive symptoms between urban and rural areas. Some studies reported that rural populations in Korea are more likely to display higher levels of depressive symptoms than urban populations (14, 44). However, many of the studies are cross-sectional or do not use national-wide samples. Park et al. (54) reported that older adults in urban areas had a mean score of 10.07 for depression, compared to a mean score of 5.82 of their rural peers. Further, in the nationwide Korean Community Health Survey, Jeong et al. (45) reported that the prevalence of depressive symptoms was 3.33% in urban areas and 2.59% in rural areas. Therefore, the findings of this study represent the health disparities between urban and rural areas.

In this study, less frequent contact with neighbors (< 1/week) or children was associated with increased depressive symptoms. We found that urban residents who had less contact with their neighbors had higher depressive symptoms than their rural counterparts. In addition, inactive labor force participation increased depressive symptoms in both urban and rural areas, consistent with previous reports that participation in social activities alleviates depressive symptoms (20, 48). Health behaviors, such as physical activity, alcohol consumption, and regular diet, are known to affect depression regardless residence areas. In the present study, urban residents who lacked regular exercise had higher rates of depressive symptoms than rural residents. Exercise is known to play a critical role in preventing depression and reducing depressive symptoms (54). Physical status may contribute to depressive symptoms (48). According to previous studies, the relationship between depression and BMI is U-shaped (55, 56), indicating that both underweight and overweight individuals had a higher incidence of depression, and we observed a higher incidence of depressive symptoms among underweight adults; however, no participant was classified as overweight (BMI ≥25 kg/m^2^) in this study. Regarding alcohol consumption, the non-drinking group had a higher incidence of depressive symptoms. Similarly, Jung (57), who analyzed the relationship between depression and drinking using KLoSA, reported that drinking lowered the depressive symptoms of older adults. In this study, we considered the current drinking status and did not investigate the drinking patterns and frequency. Thus, further studies are needed to explore the relationship between depressive symptoms and drinking patterns and frequency.

In 2020, the WHO and United Nations declared a 10-year global action plan – UN Decade of Healthy Aging (2021–2030) (58) – and established healthy aging as a global agenda. However, healthy aging is a multidimensional and highly heterogeneous concept, and there are no reliable tools or survey protocols to measure it. Recently, studies on the development of a healthy aging index have been reported (58). Therefore, we compared the incidence of healthy aging through the research results of successful aging, a similar concept that has been used more frequently (37). In the present study, the prevalence of healthy aging at baseline was 39.4%, which was slightly higher than the 32–35% reported in a previous KLoSA survey among adults over 45 years old (20). Another study comprising 2,157 participants aged 70 years and older from five European countries reported that 41.8% were healthy agers (59). Further, among 2,565 older adults aged 65 years and above in Singapore, 25.4% showed healthy aging (60). In contrast, 3,100 veterans aged over 60 years in the United States reported a self-rated healthy aging of 79% (61). These differences can be explained by the varied definitions of healthy aging and population characteristics among the above-mentioned studies. Therefore, healthy aging should be monitored using standardized healthy aging tools that consider cultural differences in various countries.

Among the five indicators of healthy aging, ADLs (unadjusted HR = 5.05) and IADLs (unadjusted HR = 3.72) demonstrated a higher risk ratio for depressive symptoms than the remaining factors. ADLs and IADLs are crucial for healthy aging because the ability to perform these activities is essential for maintaining independence and autonomy. In previous studies, ADLs and IADLs were strongly associated with depressive symptoms, even after adjusting for demographic and socioeconomic conditions (62, 63). The present study findings support that healthy aging intervention programs should include improvement of physical function as a main component (64–66). Among the indicators of healthy aging, life satisfaction constituted the second highest risk ratio for depressive symptoms (unadjusted HR = 4.27). A systematic review of healthy aging across 13 countries revealed that a positive attitude, such as satisfaction with life, was crucial for healthy aging (67). These factors should be considered in developing healthy aging interventions.

A strength of this study is that it compares the incidence of depressive symptoms over time. To achieve this, we applied the Anderson-Gill model, which considers depressive symptoms as repeated events, and not as censored data, and thereby increased the completeness of the results. Therefore, our findings may lead to a deeper understanding of the characteristics of depressive symptoms that are associated with a high risk of recurrence and suggest possible risk factors. However, although we analyzed longitudinal data, causality in the relationship between healthy aging and depressive symptoms could not be ascertained. Since exploring factors on depression using various study design, many studies have investigated determinants focusing on individual factors, including sex, income, chronic disease, and health behaviors (10, 11, 14, 23). In the present study, more urban than rural residents had healthy aging. However, we did not analyze environmental factors, such as service facilities and social capital, that could contribute to these differences. This is because the KloSA data do not include these environmental variables.

This study has some limitations. First, due to the limited data sources and the fact that this was a secondary data analysis, the analysis did not include physical environmental factors, such as housing, neighborhood facilities, and social capital that influence depressive symptoms. Physical environmental factors decrease the risk of depressive symptoms (68). Therefore, future studies should consider these factors and their effect on regional differences in the prevalence of depressive symptoms. Second, there is no clear consensus on healthy aging. A systematic review of healthy aging identified 65 models that define healthy aging in the literature, and the subcomponents vary greatly, ranging from two to nine. Moreover, some frameworks include depression as a component of healthy aging (37). In this study, healthy aging was operationalized based on successful aging, which is widely utilized as the closest concept to healthy aging and has been employed in the KLoSA study. Third, depressive symptoms were self-reported and were not ascertained through diagnostic tests. However, the CES-D is a reliable and validated screening instrument for depressive symptoms. A systematic review and meta-analysis (69) confirmed that the CES-D has acceptable screening accuracy for depression in the general population or in primary care. Nonetheless, these results should be interpreted with caution because an objective diagnostic measure of depression was not utilized. Fourth, this study lacks information on the underlying factors responsible for these differences and the mechanisms that cause these inequalities. Further research is needed to investigate the effects of various neighborhood-level factors on depressive symptoms and their interaction at the community level. Finally, the variables included in this study could also change over time. Therefore, future research should employ appropriate analysis methods to investigate how the changes in healthy aging and other variables affect the occurrence of depressive symptoms.

The present study findings provide insight into potential approaches to reducing depressive symptoms in urban and rural areas. Given that healthy aging is a crucial factor for depressive symptoms, it should be improved starting from middle age. In addition, healthy aging is related to urbanization, necessitating the need to reduce regional disparities in factors that affect healthy aging.

## 5 Conclusion

As urbanization accelerates, urban residents have a higher risk of depressive symptoms than rural residents. Healthy aging is an essential factor in reducing depressive symptoms. To achieve healthy aging, appropriate interventions and policies that target the middle-aged adults and gradually extend to older adults should be developed with reference to the various factors that cause regional and individual differences in healthy aging.

## Data availability statement

Publicly available datasets were analyzed in this study. This data can be found at: Korean Longitudinal Study of Aging (KLoSA) https://survey.keis.or.kr/eng/klosa/klosa01.jsp.

## Author contributions

SK: Conceptualization, Funding acquisition, Writing – review & editing. JH: Formal analysis, Methodology, Supervision, Writing – review & editing. DK: Formal analysis, Methodology, Writing – review & editing. BK: Conceptualization, Writing – original draft, Writing – review & editing.

## Footnotes

^1^ https://survey.keis.or.kr/eng/klosa/klosa01.jsp

^2^ https://survey.keis.or.kr/eng/klosa/klosa01.jsp
