# Supplementary material for: Regional differences in the effects of healthy aging on depressive symptoms: a Korean longitudinal study of aging (2006–2020)
Source: Front Public Health. 2024 Jan 16;12:1256368. doi: 10.3389/fpubh.2024.1256368 (PMC10824904; doi:10.3389/fpubh.2024.1256368)
Supplement: Supplementary file 1 [file Table_1.DOCX]

**Additional File Table 1.** Incidence of depressive symptoms

| **Waves** | **Variables** | **Urban** | **Rural** | ***p*-value** |
| --- | --- | --- | --- | --- |
|  |  | **n (%)** | **n (%)** |  |
| 2^nd^ wave | None | 4588 (91.1) | 1373 (88.2) | 0.001 |
|  | Depressive symptoms | 446 (8.9) | 183 (11.8) |  |
| 3^rd^ wave | None | 4140 (89.7) | 1264 (87.6) | 0.023 |
|  | Depressive symptoms | 474 (10.3) | 179 (12.4) |  |
| 4^th^ wave | Non | 3968(90.8) | 1255 (90.4) | 0.703 |
|  | Depressive symptoms | 404 (9.2) | 133 (9.6) |  |
| 5^th^ wave | None | 3679 (88.7) | 1135 (86.9) | 0.703 |
|  | Depressive symptoms | 467 (11.3) | 171 (13.1) |  |
| 6^th^ wave | None | 3433 (87.9) | 1152 (93.6) | <0.001 |
|  | Depressive symptoms | 471 (12.1) | 79 (6.4) |  |
| 7^th^ wave | Non | 3251 (88.6) | 1025 (91.4) | 0.009 |
|  | Depressive symptoms | 419 (11.4) | 97 (8.6) |  |
| 8^th^ wave | None | 3040 (88.6) | 953 (93.0) | <0.001 |
|  | Depressive symptoms | 393 (11.5) | 393 (7.0) |  |
