# Supplementary material for: Regional differences in the effects of healthy aging on depressive symptoms: a Korean longitudinal study of aging (2006–2020)
Source: Front Public Health. 2024 Jan 16;12:1256368. doi: 10.3389/fpubh.2024.1256368 (PMC10824904; doi:10.3389/fpubh.2024.1256368)
Supplement: Supplementary file 2 [file Table_2.DOCX]

**Additional File Table 2.** Effect of healthy aging on depressive symptoms -unadjusted model

| **Domains** | **Criteria** | **Variables** | **HR** | **95% CI** | ***p*-value** |
| --- | --- | --- | --- | --- | --- |
| Avoidance of disease | Number of chronic diseases | | | | |
|  | 0 | HA | [ref] | | |
|  | ≥1 | Usual aging | 2.11 | (1.99−2.26) | <0.0001 |
| Good physical function | ADL |  | | | |
|  | 0 | HA | [ref] | | |
|  | ≥1 | Usual aging | 5.05 | (1.99−2.26) | <0.0001 |
|  | IADL |  | | | |
|  | 0–1 | HA | [ref] | | |
|  | ≥2 | Usual aging | 3.72 | (3.47−3.99) | <0.0001 |
|  | Sum of ADL and IADL | | | | |
|  | 0–1 | HA | [ref] | | |
|  | ≥2 | Usual aging | 3.71 | (3.43−3.98) | <0.0001 |
| Normal cognitive  function | MMSE-K |  | | | |
|  | ≥24 | HA | [ref] | | |
|  | <24 | Usual aging | 3.80 | (3.57−4.04) | <0.0001 |
| Active social engagement | Participation in one or more activities | | | | |
|  | ≥1 | HA | [ref] | | |
|  | 0 | Usual aging | 2.77 | (2.60−2.95) | <0.0001 |
| Good psychological adaptation | Satisfaction with one’s life | | | | |
|  | ≥60 | HA | [ref] | | |
|  | <60 | Usual aging | 4.28 | (3.99−4.59) | <0.0001 |

HR, hazard ratio; CI, confidence interval; ADL, activities of daily living; IADL, instrumental ADL; MMSE-K, Mini Mental State Examination; HA, healthy ageing
